# Postinflammatory hyperpigmentation after human cold pain testing

**DOI:** 10.1097/PR9.0000000000000569

**Published:** 2016-08-23

**Authors:** Melissa J. Wolz, Katelyn E. Sadler, Caela C. Long, Daniel S. Brenner, Brian S. Kim, Robert W. Gereau, Benedict J. Kolber

**Affiliations:** a Department of Biological Sciences and Chronic Pain Research Consortium, Duquesne University, Pittsburgh, PA, USA; b Washington University Pain Center and Department of Anesthesiology, St. Louis, MO, USA; c Center for the Study of Itch, Washington University in St. Louis, St. Louis, MO, USA

**Keywords:** Cold pain, Hypersensitivity, Adverse event, Human testing, Postinflammatory hyperpigmentation

## Abstract

Changes in cold temperature sensitivity are often associated with chronic pain conditions. Progress in understanding the neurobiological mechanism underlying these changes and resulting development of effective therapies has been slowed by the accessibility and affordability of devices used to measure thermal sensitivity in humans. To address this gap, we developed an inexpensive method to measure cold pain thresholds in healthy adult volunteers using dry ice and a thermode. However, early in preliminary testing, a subject presented with epidermal postinflammatory hyperpigmentation that lasted for >200 days. Although this response was unique among the small number of subjects in development of the assay, it raised questions as to the safety of the assay design.

## Abstract 2

After assessment of thermal sensitivity during development of a novel cold pain assay, one subject developed postinflammatory hyperpigmentation. Resultant epidermal darkening gradually faded without clinical intervention.

## 1. Introduction

Increased sensitivity to cold stimuli (eg, ice, cold liquids, etc.) is a common symptom described by patients with chronic pain.^1–5^ Identification of cold pain receptors (eg, TRPA1 and TRPM8^1^) in the last few years has sparked rodent studies on the mechanisms of cold sensation^6,7^; however, the number of human studies on cold pain has remained low, in part because of available assay deficiencies.^8^

In humans, methods to assess cold sensitivity are limited to verbal reports of discomfort after cold stimuli application, the cold pressor test, or use of Peltier thermodes.^8^ All these methods have one or more limiting factors as follows: verbal reports are not quantifiable or reproducible between subjects, the cold pressor test only measures cold pain tolerance and not cold pain thresholds, and Peltier devices are cost prohibitive for wide adoption.^9,10^

Recently, a new cold sensory assay was developed for use in animals. This assay uses dry ice to consistently cool a limited area underneath an animal's foot until a withdrawal response analogous to those observed in the traditional thermal plantar assay is observed.^7,11,12^ We applied a similar approach to allow for quick and inexpensive measurement of cold sensitivity in humans. Here, we describe an unintended hyperpigmentation response that was observed during early pilot testing of this assay.

## 2. Methods

The study was performed under approval from the Duquesne University Institutional Review Board (IRB) (protocol #015-02-11 from 3/19/2015 to 3/18/2018). Case participant gave written informed consent before enrollment into the study. Exclusion criteria included history of cardiac, respiratory, neurological, or musculoskeletal disease, acute or chronic pain conditions, diabetes, anxiety or depression conditions, being currently pregnant, and allergy to hot chili pepper, benzocaine, menthol, or hand moisturizer. Three individuals completed the study before the postinflammatory hyperpigmentation (PIH) event occurred. After the adverse event described in this article, the study was suspended. The primary investigator (BJK) reported this event to the Duquesne University IRB who subsequently reported the event to the United States Office of Human Research Protections, Division of Compliance Oversight (10/6/2015).

### 2.1. Testing Apparatus

**Figure 1.**
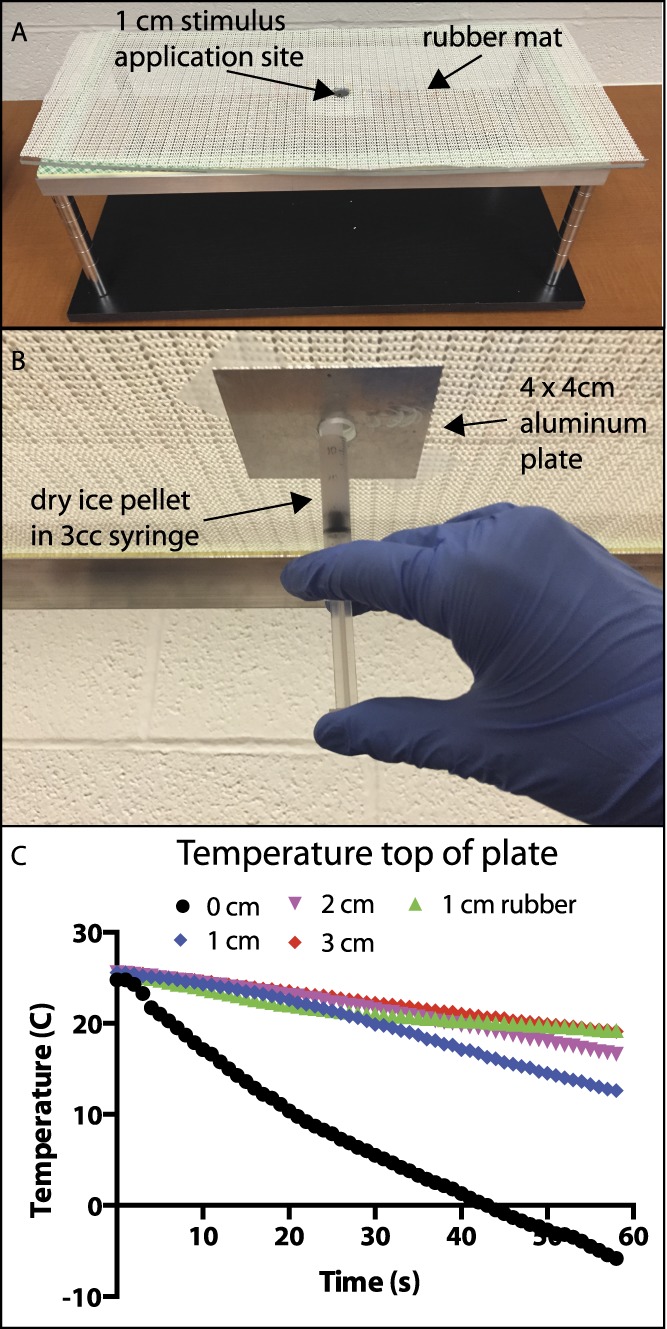
Setup of cold sensitivity assay. (A) Photograph showing platform for testing human subject. Subject's arm insulated from surface by thin rubber mat (1 mm thickness) except for stimulus application site (∼1 cm circle as indicated). (B) Photograph showing application of dry ice pellet to the underside of a 4.5 × 4.5-cm aluminum square (2.4 mm thickness). (C) Temperature measured at the interface of the aluminum plate and a piece of styrofoam at various distances from center during dry ice application without rubber mat on platform. The “1-cm rubber” measurement was made on top of the rubber mat 1 cm from stimulus center to demonstrate that the skin that is resting on the rubber mat (>1 cm from stimulus center) is insulated from the primary cooling effect of the dry ice application. For all trials involving human subjects, 0^o^C was defined as the safety cutoff.

This new assay was modeled on the cold plantar assay developed for use in rodents.^11,13^ A Plexiglas platform (55.8 × 24.4 cm) was suspended 20.3 cm above the counter surface with support bars from a standard shelving unit (Fig. 1A). A 1-cm hole was drilled into the center of the Plexiglas platform. A 4.5 × 4.5-cm (2.4 mm thick) aluminum square was centered over hole in Plexiglass and secured in place. A rubber mat (1 mm thick) was placed on top of Plexiglass covering entire surface except for a ∼1-cm hole that was centered on the aluminum square. The aluminum square was cooled through application of a packed dry ice pellet to the underside of the aluminum plate (opposite side from subject) (Fig. 1B). The dry ice pellet was made as previously described.^11^ A low-profile type K thermocouple (1 mm width and 0.5 mm thick; Model FLUKE 54 IIB; Fluke Corporation Everett, WA with reported temp accuracy of ± 0.05% + 0.3°C) was taped in place directly next to the center of the aluminum square. The thermode was selected to minimize interference of stimulus presentation and could not be detected through forearm touch alone. The thermode was used to monitor, in real-time, the temperature of the interface between the metal and the subject's skin. To evaluate the temperature that would be experienced by a subject during a trial, multiple test trials were completed using a 25 cm L × 25 cm W × 3 cm H Styrofoam block over the aluminum square with or without the rubber mat in place. The center of the aluminum plate (application site) cooled the most rapidly (Fig. 1C).

### 2.2. Study design (for case subject)

The subject received 2 treatments, 10% menthol cream (CVS Muscle Rub; CVS Pharmacy, Woonsocket, RI) or 20% topical benzocaine (Lanacane Maximum Strength Spray; Lanacane, Reckitt Benckiser, Slough, United Kingdom), in a double-blind crossover design compared with vehicle (Aveeno daily moisturizing lotion for menthol; 24% ethanol in H_2_O for benzocaine). Although the data are not presented here, we hypothesized that menthol (a TRPM8 agonist) would increase cold sensitivity and that benzocaine (local anesthetic) would decrease cold sensitivity. Briefly, on each day of the experiment, the subject received 2 rounds of cold testing, each test consisting of one baseline trial followed by a treatment or vehicle trial per stimulation site. Stimulation sites were 2.5 cm squares traced on the subject's forearm with a marker. Three stimulation sites were spaced 2.5 cm apart from each other and were positioned roughly along the T1 dermatome on each forearm. The time between individual trials was 2 minutes, and the experimenter alternated between the left and right forearm stimulation sites. The total number of cold tests per stimulation square was 4 (2 per day). After testing on both days, subject was allowed to wash both forearms.

## 3. Results

During the development of the cold cutaneous assay (Fig. 1), one subject experienced an adverse response to cold testing. The subject was a 26-year-old woman of European descent with Fitzpatrick scale type III skin coloration, possessing no study exclusion criteria. The subject had an average withdrawal temperature of 11.1 ± 4.5°C in the left forearm and 11.8 ± 6.9°C in the right forearm. Initially, the subject experienced normal responses to cold stimuli (red marks in testing sites) and did not report any adverse sensitivity after completion of testing.

**Figure 2.**
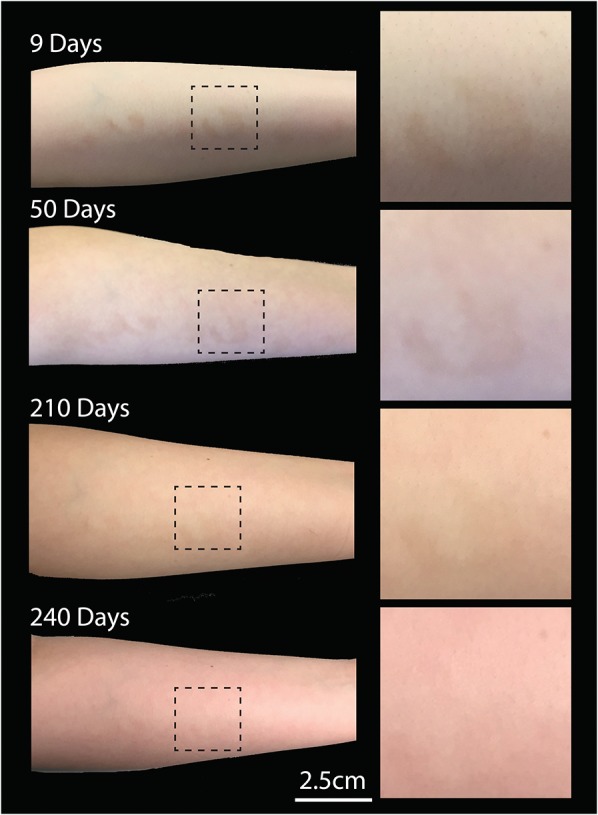
Case example of postinflammatory hyperpigmentation. Case subject reported persistent skin pigmentation that was first noticed 9 days after end of experiment. Subject showed pigmentation on both the left and right (not shown) forearms on all 3 stimulation sites per arm. Dotted box is centered on the same stimulation site for photographs taken on days 9, 50, 210, and 240 after the end of the experiment. Dotted box area is enlarged to the right of each image to show example hyperpigmentation and the gradually lightening of hyperpigmentation with time.

Although other study participants also reported red skin discoloration in stimulation sites, these marks typically faded over the course of one day. Nine days after testing, however, the case subject presented with 6 crescent-shaped macules of varying color on both forearms, each one developing around the site of stimulus application (Fig. 2). The pigmentation areas were flat. The subject did not report changes in pain sensitivity, touch sensitivity, itch, or thermal allodynia (eg, from a hot shower), although no quantitative testing was performed before or during the development of hyperpigmentation because of IRB-approved protocol limitations. This pigmentation was diagnosed as PIH by a board-certified dermatologist (BSK) on clinical examination. Fifty days after testing, the marks were lighter but still visible. By 240 days after testing and without any therapeutic interventions, dark macules had faded to visible but near-normal pigment macules.

## 4. Discussion

Here, we report the development of PIH at 6 sites on a subject's left and right forearms after cold sensitivity testing. The hyperpigmentation developed around the stimulus borders where the skin temperature was several degrees warmer than that in the center, which we believe was likely ischemic. Our hypothesis is that the subcutaneous blood flow was likely increased because of inflammation at these borders, leading to the curious size and shape of the hyperpigmented macules (Fig. 2), corresponding to the outer borders of the stimulus.

It is unlikely that the pigmentation is due to skin freezing; an ultraprecise thermocouple was used to monitor temperature at the skin surface with a 0^o^C safety cutoff. Because the subject withdrew her arms at ∼11^o^C, skin freezing is an unlikely explanation for the skin reaction, although we cannot rule out a potential overestimation of the temperature by the thermocouple. Another subject received the same treatment and similar sensitivity thresholds as the case subject with no adverse events reported (data not shown). In addition, contact dermatitis induced by the aluminum plate or treatments/vehicles may have induced PIH. Again, we think this is unlikely due to the shape of the hyperpigmented regions and the fact that pigmentation was equally induced on both arms although each arm received a different treatment/vehicle.

Postinflammatory hyperpigmentation is commonly associated with acne vulgaris, contact and atopic dermatitis, psoriasis, laser hair removal, and chemical/thermal burns.^14,15^ Inflammation stemming from these conditions results in the local release of chemical mediators including prostaglandins and leukotrienes, arachidonic acid metabolites that regulate the activity of tyrosinase, the melanocyte-localized enzyme that is responsible for melanin synthesis.^16^ The pigmentation in this case was indicative of either epidermal melanosis where excess melanin is endocytosed by resident keratinocytes^17^ or dermal melanosis where excess melanin is endocytosed by resident macrophages.^18^ Lack of a skin biopsy at the time of diagnosis prevents further mechanistic clarity for this specific case. Because of increased basal levels of melanin, PIH more commonly affects individuals with darker skin tones (ie, skin phototypes III to VI on the Fitzpatrick scale; typical of individuals of Mediterranean, Middle Eastern, or African descent)^19^ than those with lighter skin (ie, skin phototypes I–II; typical of individuals of European descent).^20^

Reports suggest that individual variability in melanocyte inflammatory responses is also likely to determine a person's susceptibility to PIH.^21^ In this report, the affected individual self-reported having type III skin, citing occasional sunburns and the ability to maintain a multimonth tan after prolonged sun exposure. Anecdotal reports like this could be useful exclusionary statements for individuals who would otherwise be included in future cold cutaneous assay experiments. The cold cutaneous assay we presented here still needs to be validated as a reliable, accurate measure of cold sensitivity. Our intent in this case report is not to establish this model but instead to serve as a cautionary tale for future development of similar assays.

## Conflict of interest statement

The authors have no conflicts of interest to declare.
